# Characterization of the complete mitogenome data of *Ischyja marapok* (Lepidoptera: Noctuoidea: Erebidae) from Malaysia

**DOI:** 10.1016/j.dib.2023.109253

**Published:** 2023-05-21

**Authors:** Marylin Miga, Puteri Nur Syahzanani Jahari, Sivachandran Parimannan, Heera Rajandas, Muhammad Abu Bakar Abdul-Latiff, Yap Jing Wei, Mohd Shahir Shamsir, Faezah Mohd Salleh

**Affiliations:** a Department of Biosciences, Faculty of Science, Universiti Teknologi Malaysia, Johor Bahru, Johor 81310, Malaysia; b Centre of Excellence for Omics-Driven Computational Biodiscovery (COMBio), Faculty of Applied Sciences, AIMST University, Bedong, Kedah 08100, Malaysia; c Environmental Management and Conservation Research Unit (ENCORE), Faculty of Applied Sciences and Technology (FAST), Universiti Tun Hussein Onn Malaysia, Pagoh Higher Education Hub, Muar, Johor 84600, Malaysia; d Centre of Research for Sustainable Uses of Natural Resources (SUNR), Faculty of Applied Sciences and Technology (FAST), Universiti Tun Hussein Onn Malaysia, Pagoh Higher Education Hub, Muar, Johor 84600, Malaysia

**Keywords:** Mitochondrial genome, Annotation, Assembly, Phylogenetic analysis, Moth, Erebidae

## Abstract

*Ischyja marapok* is a moth species from the genus *Ischyja,* a member of the Lepidoptera family, Erebidae. Due to their wide variation, this family constitutes the largest described species, however, the mitogenome dataset on the genus *Ischyja* is scarce. Hence, the mitochondrial genome dataset of *Ischyja marapok* from Malaysia was completely sequenced using the next-generation sequencing technology, Illumina NovaSeq 6000 and analyzed. The mitogenome has a sequence length of 15,421 bp, consisting of 13 protein-coding genes (PCGs), 22 transfer RNAs (tRNAs), 2 ribosomal RNAs (rRNAs) and a control region. The mitogenome is *A* + *T* biased (80.6%), with the base composition of A (39.2%), T (41.4%), C (11.9%) and G (7.5%). Among the 13 PCGs, 12 were initiated by the standard ATN codon, except for COX1 which utilizes the CGA start codon. Two PCGs were terminated with an incomplete stop codon T, while others ended with a TAA codon. Phylogenetic tree analyses showed that the sequenced *I. marapok* resides within the Erebinae subfamily and is closely related to *Ischyja manlia* (MW664367) with high bootstrap support and posterior probabilities. This dataset presented the mitogenome data of *I. marapok* from Malaysia, which is valuable for further research of their phylogeny and the diversification of the *Ischyja* genus. Also, this dataset can be implemented and used as references to assess environmental changes in the terrestrial ecosystem via environmental DNA approaches. The mitogenome of *I. marapok* is available in GenBank under the accession number ON165249.

**Specifications Table**

**Table:** 

Subject	Genomics
Specific subject area	Lepidoptera, Noctuoidea, Mitogenomics
Type of data	Tables: Sequencing data of *I. marapok* mitogenome, features of *I. marapok* mitogenome, base composition and AT/GC skew, list of Lepidoptera mitogenomes used for the phylogenetic analysis Figures: Circular mitogenome map, features of the control region, phylogenetic tree analysis Fasta: Mitogenome sequence data
How the data were acquired	Sequencing: Illumina NovaSeq 6000 with 150 paired-end mode (PE150). Mitogenome assembly: MITOS2 web server and PALEOMIX BAM pipeline. Mitogenome annotation: MITOS v2 web server and the Open Reading Frame (ORF) Finder. Circular mitogenome map: OGDraw. Phylogenetic analyses: IQ-Tree and MrBayes v3.2.7 programs were used to build the phylogenetic trees using Maximum-Likelihood (ML) and Bayesian Inference (BI) probability methods. Phylogenetic tree visualization: Figtree v1.4.4.
Data format	Raw and analyzed
Description of data collection	Genomic DNA: Fresh tissue sample of *I. marapok* using the Qiagen Blood and Tissue Kit (Qiagen, Valencia, CA) prior to fragmentation using a Bioruptor® system. Library preparation: NEBNext® Ultra™ II DNA Library Prep Kit for Illumina®.
Data source location	• Location: Ayer Hitam Forest Reserve, Johor. • Town: Muar, Johor • Country: Malaysia • Latitude and longitude for collected samples: 2.03 N 102.49 E
Data accessibility	Repository name: NCBI BioProject Data identification number: PRJNA753627 Direct URL to data: http://www.ncbi.nlm.nih.gov/bioproject/753627 Repository name: NCBI BioSample Data identification number: SAMN20720551 Direct URL to data: https://www.ncbi.nlm.nih.gov/biosample/20720551 Repository name: NCBI GenBank Data identification number: ON165249 Direct URL to data: https://www.ncbi.nlm.nih.gov/nuccore/ON165249 Repository name: Mendeley Data Data identification number: 10.17632/zn4b8sgcyk.1 Direct URL to data: https://data.mendeley.com/datasets/zn4b8sgcyk

## Value of the Data

• The mitogenome data presented here provides the complete and novel mitochondrial genome of *I. marapok* from the Lepidoptera family Erebidae, originating from Malaysia.
• This dataset provides useful information for other researchers who are working on assembling and annotating the mitogenomes of Erebidae species.
• The provided dataset can also be used to further analyze the phylogenetic relationships of the Erebidae family and the phylogenetic position of the *Ischyja* genus.
• Additionally, as one of the bioindicator species, the mitogenome data provided here will also benefit researchers who are working on the application of environmental DNA (eDNA) for biodiversity monitoring via DNA approaches.

## 1 Objective

*Ischyja marapok* is a moth species from the family Erebidae, the largest family in Noctuoidea. Due to the advancement in next-generation sequencing technologies, there are approximately 220 complete mitogenome sequences of Erebidae species published in the NCBI database, however, the mitogenome data for the genus *Ischyja* is scarce. Additionally, the genus *Ischyja* has been placed under *incertae sedis* in the family Erebidae and is in need of more sampling to improve their placement within the family [1,2]. To date, only one complete mitogenome data has been reported in NCBI database for this genus originating from India [1], however, none has been reported from Malaysia. Therefore, this work aims to generate and characterize the complete mitogenome of *I. marapok* originating from Malaysia, as well as their phylogenetic position in Erebidae.

## 2 Data Description

**Fig. 1:**
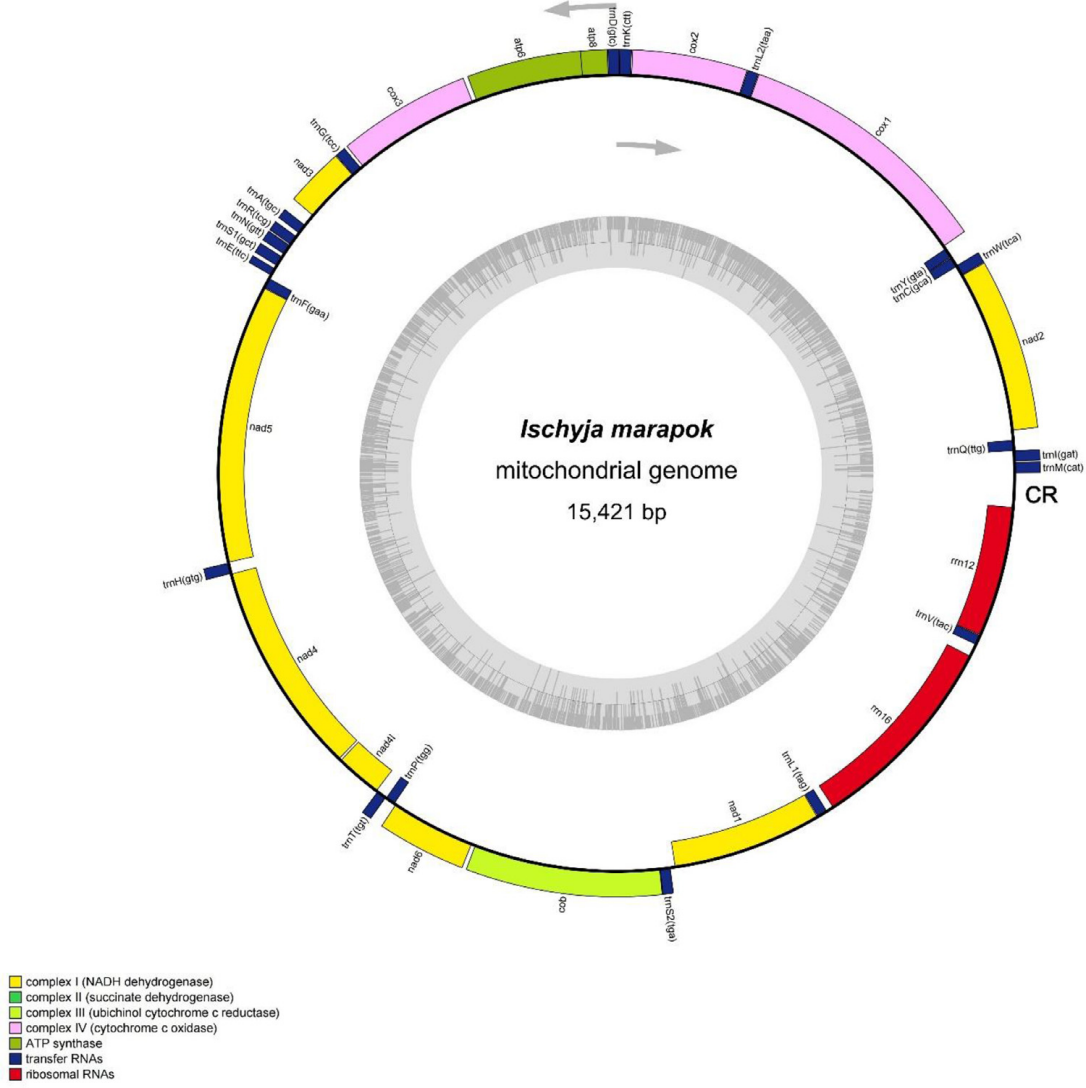
A circular mitogenome map of *I. marapok* originating from Malaysia as generated by OGDraw [3]. The outer circle area indicates heavy strand, while the inner circle indicates the light strand. The arrows represent the direction of transcription, and the inner gray ring area expresses the mitogenome GC content. CR represents the control region.

The complete mitochondrial genome of *I. marapok* is 15,421bp in length, comprising of 13 protein-coding genes (PCGs), 22 transfer RNAs (tRNAs), 2 ribosomal RNAs (rRNAs) and a control region (Fig. 1). Using the Illumina NovaSeq 6000 sequencing technology, we managed to obtain a total of 10,122,328 raw reads with the final mitogenome displaying a depth of coverage 109.5X (Table 1). The mitogenome is *A*+ *T* biased (80.6%) with a nucleotide composition of A (39.2%), T (41.4%), C (11.9%) and G (7.5%) (Table 2). Nucleotide composition of the whole mitogenome showed high occurrence of T over A, and C over G, giving rise to the AT-skew of −0.027 and GC-skew of −0.227. Similar occurrence was also found in the control region where there is more T over A, and C over G.

**Table 1:** Sequencing data of *I. marapok* mitogenome.

	*Ischyja marapok*
Raw reads	10,122,328
Trimmed reads	10,122,304
Ave. read length	149.6
Mapped reads	11,281
% mapped reads	0.001
Depth of coverage (X)	109.5

**Table 2:** Base composition and AT/GC skewness for each gene region of *I. marapok* mitogenome.

Gene	Size (bp)	A%	G%	T%	C%	*A* + T%	AT skew	GC skew
Whole mitogenome	15,421	39.2	7.5	41.4	11.9	80.6	−0.027	−0.227
Protein coding	11,214	33.9	10.5	45.3	10.2	79.2	−0.144	0.014
tRNA	1458	41.8	10.6	39.0	8.5	80.9	0.035	0.110
rRNA	2097	43.3	11.1	40.4	5.2	83.7	0.035	0.362
Control region	208	43.3	1.4	51.9	3.4	95.2	−0.090	−0.417

The mitogenome has a gene order of *trnM-trnI-trnQ,* located between the control region and NAD2, which has been observed in most Lepidoptera mitogenomes. The 13 protein-coding gene sequences has a total length of 11,214bp, while the transfer RNAs are 1458bp. The length of the 12S and 16S rRNAs are 821bp and 1276bp, respectively. Most of the genes are located on the heavy strand, compared to the light strand. On the heavy strand, 9 PCGs (COX1, COX2, COX3, NAD2, NAD3, NAD6, ATP8, ATP6, CYTB) and 15 tRNAs (trnM, trnI, trnW, trnL2, trnK, trnD, trnG, trnA, trnR, trnN, trnS1, trnE, trnT, trnS2, trnH) were observed. Subsequently, the light strand places 7 PCGs (NAD1, NAD4, NAD4l, NAD5), 6 tRNAs (trnQ, trnC, trnY, trnF, trnP, trnL1, trnV) and 2 rRNAs (12S and 16S). Out of the 13 PCGs, 12 were initiated by the standard ATN start codon (ATT, ATG, ATA), except for COX1, which utilized the CGA codon. Two PCGs (COX1 and NAD4) were terminated by an incomplete stop codon, T, while the rest of the PCGs ended with a TAA stop codon (Table 3).

**Table 3:** Features of *I. marapok* mitogenome. The direction of each genes are indicated by F (forward) and R (reverse).

	Position				
Gene (anticodon)	Start	Stop	Direction	Size	Start/Stop codon
trnM(cat)	1	69	F	69	
trnI(gat)	74	140	F	67	
trnQ(ttg)	138	206	R	69	
NAD2	264	1277	F	1014	ATT/TAA
trnW(tca)	1278	1346	F	69	
trnC(gca)	1339	1406	R	68	
trnY(gta)	1407	1471	R	65	
COX1	1488	3015	F	1528	CGA/T
trnL2(taa)	3016	3083	F	68	
COX2	3084	3764	F	681	ATG/TAA
trnK(ctt)	3767	3837	F	71	
trnD(gtc)	3837	3905	F	69	
ATP8	3906	4067	F	162	ATC/TAA
ATP6	4061	4738	F	678	ATG/TAA
COX3	4762	5550	F	789	ATG/TAA
trnG(tcc)	5565	5630	F	66	
NAD3	5631	5984	F	354	ATT/TAA
trnA(tgc)	6059	6122	F	64	
trnR(tcg)	6147	6210	F	64	
trnN(gtt)	6215	6281	F	67	
trnS1(gct)	6300	6365	F	66	
trnE(ttc)	6385	6434	F	50	
trnF(gaa)	6454	6520	R	67	
NAD5	6521	8266	R	1746	ATT/TAA
trnH(gtg)	8267	8336	F	70	
NAD4	8337	9675	R	1339	ATG/T
NAD4l	9686	9979	R	294	ATG/TAA
trnT(tgt)	9985	10,049	F	65	
trnP(tgg)	10,050	10,114	R	65	
NAD6	10,122	10,652	F	531	ATT/TAA
CYTB	10,674	11,834	F	1161	ATA/TAA
trnS2(tga)	11,833	11,899	F	67	
NAD1	11,927	12,865	R	939	ATG/TAA
trnL1(tag)	12,867	12,934	R	68	
16S rRNA	12,976	14,251	R	1276	
trnV(tac)	14,328	14,391	R	64	
12S rRNA	14,392	15,212	R	821	
D - loop	15,214	15,421	F	208	

**Fig. 2:**
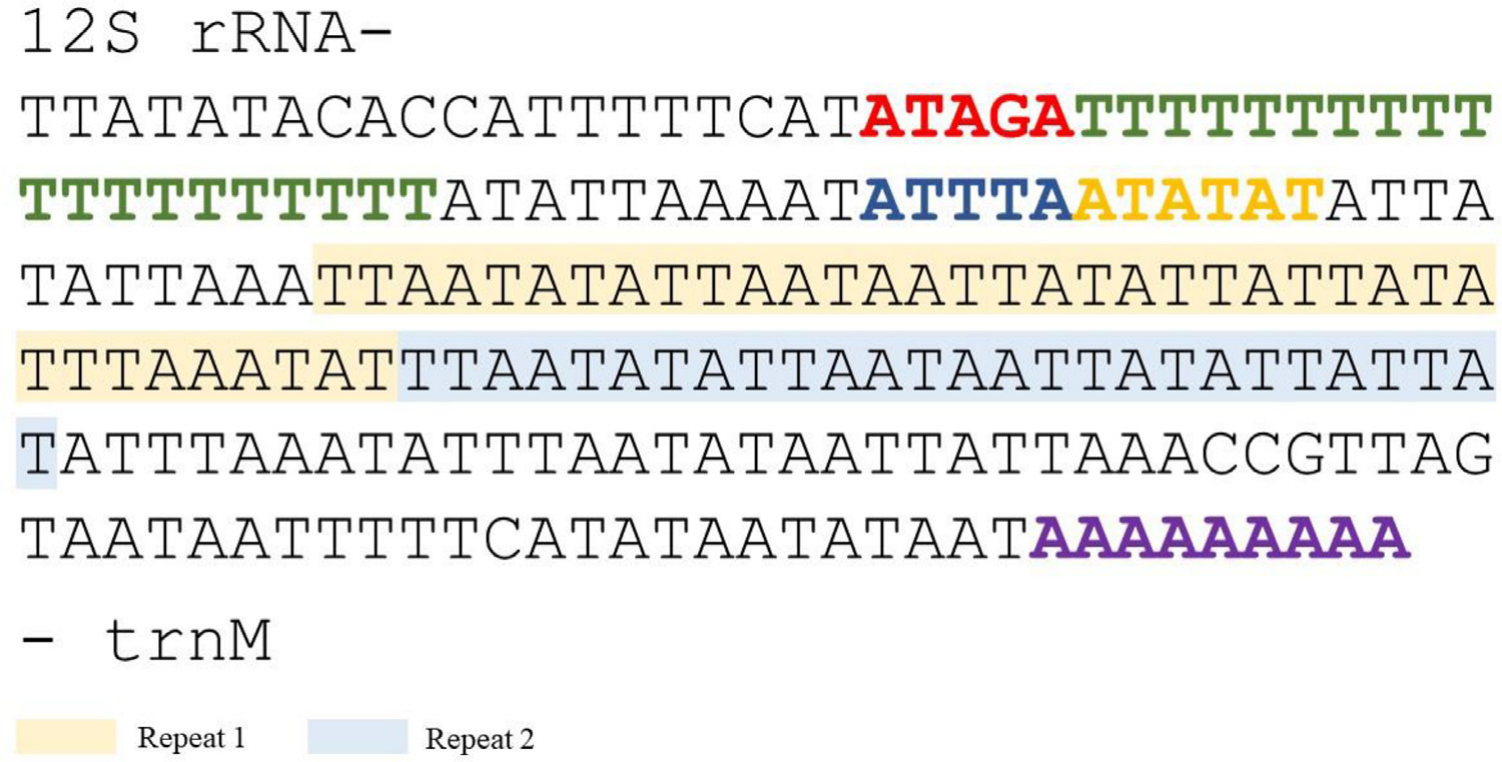
Features of the control region of *I. marapok* mitogenome. The color indicators represent the conserved motifs ‘ATAGA’ (red) and ‘ATTTA’ (dark blue); poly-T stretch (dark green), microsatellite elements AT(n) (orange), and poly-A stretch (purple). Tandem repeats are highlighted in light orange and blue color.

The control region of *I. marapok*, also known as the AT-rich region is located between 12S rRNA and trnM, spanning a total length of 208 bp. Conserved motif ‘ATAGA’ was detected close to the 12S rRNA, followed by a 20 bp poly-T stretch and microsatellite-like elements AT after the motif ‘ATTTA’. A string of poly-A was also detected up-stream of trnM. Additionally, two tandem repeats were detected as shown in Fig. 2.

**Fig 3:**
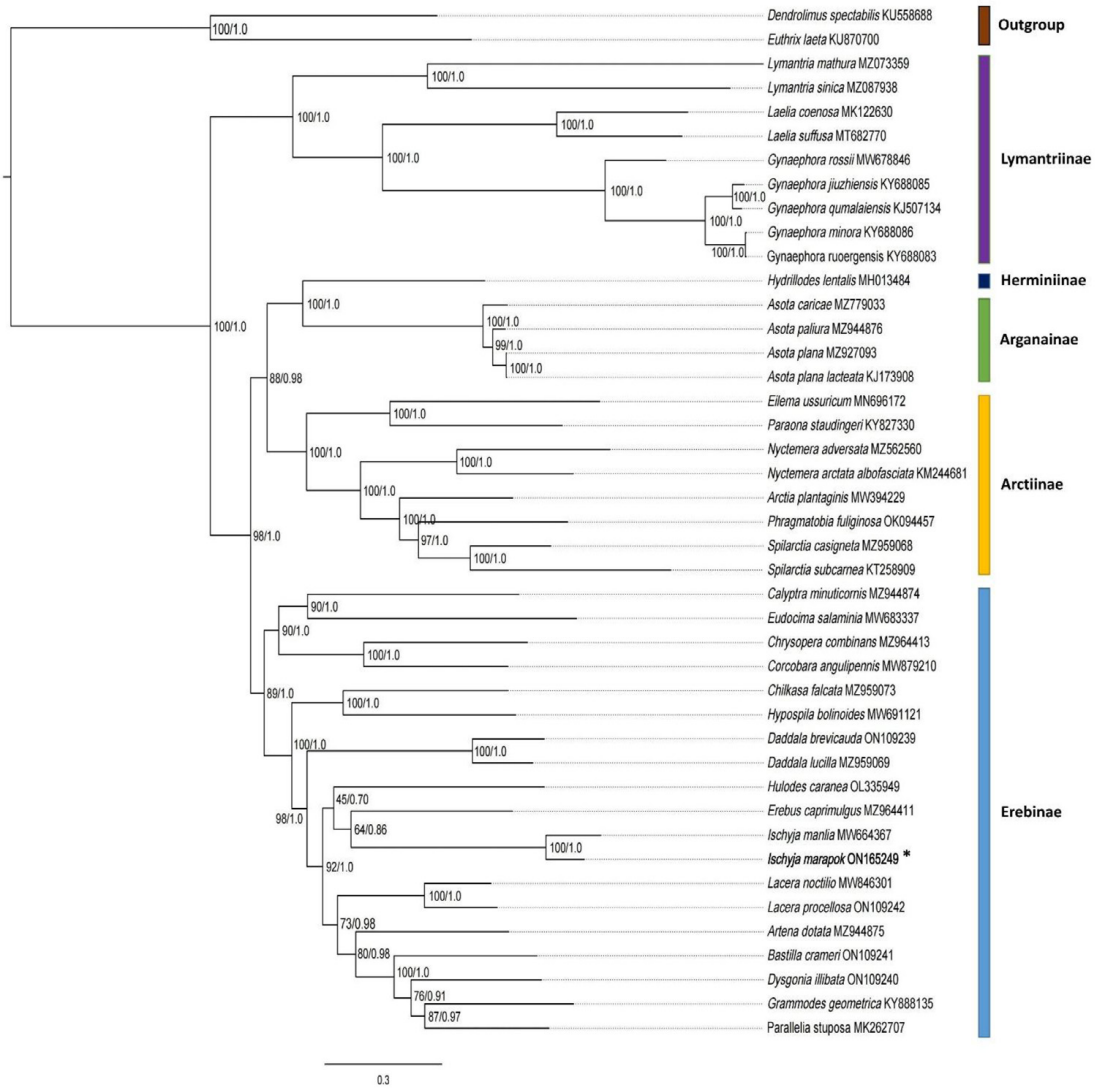
Phylogenetic tree analyses of *I. marapok* (ON165249), indicated by an asterisk (*) and 42 other Lepidopteron mitogenomes built using Maximum-Likelihood (ML) and Bayesian Inference (BI). Nodal values represent the bootstrap support (BS) and posterior probabilities (PP). *Dendrolimus spectabilis* (KU558688) and *Euthrix laeta* (KU870700) from the family Lasiocampidae were used as outgroups. The color codes located at the right side of the phylogenetic tree indicates the different subfamilies in Erebidae.

Phylogenetic analyses based on Maximum-Likelihood (ML) and Bayesian Inference (BI) were performed to determine the phylogenetic position of *I. marapok* in the Erebidae family. Thirteen concatenated protein-coding genes from 43 Lepidoptera mitogenomes (including the newly sequenced *I. marapok*) were used in the analysis (Table 4). Based on the data generated, both Maximum-Likelihood (ML) and Bayesian Inference (BI) analyses yielded identical tree topology, but with different branch length (ML= 0.3, BI=0.4). Based on Fig. 3, the newly sequenced *Ischyja marapok* in this work is clustered within the Erebinae subfamily, and is phylogenetically closer to *Ischyja manlia* (MW664367) with high bootstrap value (ML=100%), and posterior probabilities (PP=1.0). A BLAST analysis was conducted on *I. marapok* (ON165249) and *I. manlia* (MW664367) mitogenome which showed that *I. marapok* is 94.72% similar to *I. manlia* (MW664367) deposited in NCBI GenBank. Additionally, a BLAST analysis was also conducted on the COX1 sequence of *I. marapok* (ON165249) with other available COX1 sequences of similar species in the database (GenBank and BOLD) and the analysis showed between 99.39% to 99.85% similarities.

**Table 4:** List of the Lepidoptera mitogenomes used to perform the phylogenetic analyses. The newly sequenced *I. marapok* is highligted in bold.

Family	Subfamily	Species	GenBank Accession No.
Erebidae	Aganainae	*Asota plana lacteata*	KJ173908
		*Asota caricae*	MZ779033
		*Asota plana*	MZ927093
		*Asota paliura*	MZ944876
	Arctiinae	*Nyctemera arctata albofasciata*	KM244681
		*Spilarctia subcarnea*	KT258909
		*Paraona staudingeri*	KY827330
		*Eilema ussuricum*	MN696172
		*Arctia plantaginis*	MW394229
		*Nyctemera adversata*	MZ562560
		*Spilarctia casigneta*	MZ959068
		*Phragmatobia fuliginosa*	OK094457
	Erebinae	*Grammodes geometrica*	KY888135
		*Parallelia stuposa*	MK262707
		*Ischyja manlia*	MW664367
		*Eudocima salaminia*	MW683337
		*Hypospila bolinoides*	MW691121
		*Lacera noctilio*	MW846301
		*Corcobara angulipennis*	MW879210
		*Calyptra minuticornis*	MZ944874
		*Artena dotata*	MZ944875
		*Daddala lucilla*	MZ959069
		*Chilkasa falcata*	MZ959073
		*Erebus caprimulgus*	MZ964411
		*Chrysopera combinans*	MZ964413
		*Hulodes caranea*	OL335949
		*Daddala brevicauda*	ON109239
		*Dysgonia illibata*	ON109240
		*Bastilla crameri*	ON109241
		*Lacera procellosa*	ON109249
	Herminiinae	*Hydrillodes lentalis*	MH013484
	Lymantriinae	*Gynaephora qumalaiensis*	KJ507134
		*Gynaephora ruoergensis*	KY688083
		*Gynaephora jiuzhiensis*	KY688085
		*Gynaephora minora*	KY688086
		*Laelia coenosa*	MK122630
		*Laelia suffusa*	MT682770
		*Gynaephora rossii*	MW678846
		*Lymantria mathura*	MZ073359
		*Lymantria sinica*	MZ087938
	Unassigned	***Ischyja marapok***	ON165249

Lasiocampidae		*Dendrolimus spectabilis*	KU558688
		*Euthrix laeta*	KU870700

## 3 Experimental Design, Materials and Methods

### 3.1 Sampling, DNA extraction and data pre-processing

The adult sample of *I. marapok* (voucher no: DIM052) was collected from Ayer Hitam Forest Reserve Johor, Malaysia (2.03N 102.49 E). Genomic DNA was extracted from the hind leg tissue using the Qiagen Blood and Tissue Kit (Qiagen, Valencia, CA) prior to fragmentation using a Bioruptor® system (https://www.diagenode.com/en/categories/bioruptor-shearing-device). The library was prepared using the NEBNext® Ultra™ II DNA Library Prep Kit for Illumina®, following the manufacturer's instructions and was sent for sequencing using the Illumina NovaSeq 6000 with paired-end mode 150. The raw reads were firstly assessed for its quality using the fastQC program (https://www.bioinformatics.babraham.ac.uk/projects/fastqc/) and were trimmed for sequencing adapters using AdapterRemoval v2.3.2 [4]. The trimmed reads displayed a quality score of more than 24, thus retained.

### 3.2 Mitogenome assembly, annotation and data analysis

Using the seed input from BOLD public data (Sequence ID: LEPKA953–09.COI-5P) as reference, the mitogenome was successfully assembled by NOVOPlasty v.4.2 [5] program and run through the PALEOMIX BAM pipeline [6] (default parameters), to assess the mitogenome mapping. Next, the mitogenome annotation was performed using the MITOS v2 web server [7]. To improve the annotation, the predicted proteins were verified using the Open Reading Frame (ORF) Finder (https://www.ncbi.nlm.nih.gov/orffinder/) server, followed by alignment visualization in Jalview 2 v11.1.4 [8]. Subsequently, Tablet software [9] was used to manually check for indels and sequence coverage. To determine the total base composition, BioEdit software [10] was used by integrating the formula: AT skew= (A-T)/(*A*+ *T*) and GC skew=(G-C)/(G + C). The circular mitogenome map of *I. marapok* was generated by OGDraw [3]. Tandem repeats at the control region were predicted using the Tandem Repeats Finder tool (https://tandem.bu.edu/trf/basic_submit) (basic parameter).

### 3.3 Phylogenetic analyses

Forty-two available Lepidoptera mitogenomes from the family Erebidae and Lasiocampidae (Superfamily Bombycoidea) were downloaded from NCBI GenBank (Table 4), in which the ingroups consist of representatives from the five recognized subfamilies in Erebidae [2]. *Dendrolimus spectabilis* (KU558688) and *Euthrix laeta* (KU870700) from the family Lasiocampidae were used as outgroups. Prior to phylogenetic analyses, the protein-coding genes of each Lepidoptera mitogenomes were firstly extracted using PhyloSuite v1.2.2 [11] and aligned using MAFFT [12]. Next, ambigous sites from the 13 protein-coding genes were removed by Gblocks with default settings [13] and were concatenated. Here, PartitionFinder v2.1.1 was utilized to determine the best-partitioning schemes for the dataset [14]. For Maximum-Likelihood (ML), the analysis was performed using IQ-Tree web server [15] with 5000 ultrafast bootstrapping and the best fit model was determined by ModelFinder [16]. The Bayesian Inference (BI) tree was built using MrBayes [17], carried out for 10,000,000 generations with 4 chains, sampled every 1000 generations until the average standard deviation of split frequencies are less than 0.01. Tracer v1.7.2 was used to ensure sufficient parameter sampling and that the Estimated Sample Size (ESS) is more than 200 [18]. The resulting trees were visualized using Figtree v1.4.4 (http://tree.bio.ed.ac.uk/software/figtree/).

## Ethics Statements

No data were collected involving any human subjects, animal experiments and social media platforms.

## CRediT Author Statement

**Marylin Miga:** Conceptualization, Methodology, Data Curation, Software, Validation, Writing- Original draft preparation; **Puteri Nur Syahzanani Jahari:** Data Curation, Conceptualization, Methodology, Software, Validation, Writing- Review & Editing; **Sivachandran Parimannan:** Formal analysis, Resources, Funding acquisition; **Heera Rajandas:** Formal analysis, Resources, Funding acquisition; **Muhammad Abu Bakar-Latiff:** Resources, Funding acquisition; **Yap Jing Wei:** Resources, Funding acquisition; **Mohd Shahir Shamsir:** Methodology, Formal analysis, Resources, Funding acquisition; **Faezah Mohd Salleh:** Conceptualization, Methodology, Resources, Writing- Review & Editing, Supervision, Funding acquisition. All authors have read and approved the manuscript.

## Declaration of Competing Interest

The authors declare that they have no known competing financial interests or personal relationships that could have appeared to influence the work reported in this paper.

## Data Availability

Characterization of the complete mitogenome data of Ischyja marapok (Lepidoptera: Noctuoidea: Erebidae) from Malaysia (Original data) (Mendeley Data).
